# Application of Aluminum Oxide Nanoparticles in* Aspergillus terreus* Cultivations: Evaluating the Effects on Lovastatin Production and Fungal Morphology

**DOI:** 10.1155/2019/5832496

**Published:** 2019-01-13

**Authors:** Tomasz Boruta, Marcin Bizukojc

**Affiliations:** Lodz University of Technology, Faculty of Process and Environmental Engineering, Department of Bioprocess Engineering, Ul. Wolczanska 213, 90-924 Lodz, Poland

## Abstract

Aluminum oxide nanoparticles were supplemented to* Aspergillus terreus* ATCC 20542 precultures and the outcomes of the process were evaluated relative to the results of microparticle-enhanced and standard cultivations. The selected morphological parameters of fungal pellets (projected area, elongation, convexity, and shape factor) were monitored throughout the experiment, together with biomass, lactose, and lovastatin concentration. The qualitative and quantitative chemical analysis was performed with the use of liquid chromatography coupled with high resolution mass spectrometry. The results of the study indicated that the application of nanoparticles was indeed associated with morphological consequences, most notably the decreased pellet size. However, it turned out that the term “nanoparticle-enhanced cultivation” could not be used in the context of lovastatin production, as no marked increase of product titer was observed in nanoparticle-influenced variants relative to standard and microparticle-enhanced cultivation. In addition, the concentration of biomass in the nanoparticle-influenced runs was relatively low. Comparative analysis of total ion chromatograms revealed the presence of a molecule of unknown structure that could be detected solely in broths from standard and microparticle-containing cultures. This study represents the first evaluation of nanoparticles as the tools of morphological engineering aimed at enhanced lovastatin biosynthesis in* A. terreus* cultures.

## 1. Introduction

The remarkable biosynthetic capabilities of filamentous fungi are of central importance to microbiology and biotechnology, especially in the context of industrial manufacturing of chemicals, e.g., organic acids, enzymes, and secondary metabolites [1, 2]. Even though the molecules of microbial origin have been studied for decades both in industry and academia, it is believed that the rich biosynthetic catalogue of fungi remains mostly unexplored and harbors a myriad of potentially useful compounds. The elucidation of secondary metabolic pathways and products is far from trivial, as it often requires sophisticated methods and experimental creativity [3–9]. The studies carried out at the International Space Station, involving fungal species isolated form the Chernobyl disaster area, are the examples of recent scientific endeavors that may lead to discovering novel fungal molecules of biotechnological relevance [10]. It should be remembered, however, that even if such compounds are revealed, there is always a great deal of bioprocess-related work that needs to be performed before an effective and economically feasible industrial production can be established. This is where bioreactor process design and optimization come into play. The submerged cultivation of filamentous fungi is associated with numerous engineering challenges, e.g., dealing with highly viscous fermentation broths [11]. Importantly, it has been realized that the biosynthetic performance of these organisms is largely dependent on their morphology, namely, whether the cultivated fungus proliferates in the form of loose mycelia, clumps, or pellets [12]. What complicates the picture is the lack of general recommendations regarding the preferred morphological forms that lead to satisfactory product yields and titers. In fact, each cultivation process ought to be considered individually in order to characterize the biosynthetic performance of the employed strain as a function of observed morphological parameters. In this context, controlling the morphological evolution of mycelia is still regarded as one of the key elements of fungal bioprocess development [13–16]. Morphological engineering involves a variety of methods aimed at improving strain performance via influencing morphology. Microparticle-enhanced cultivation is one of the most frequently applied and successful approaches [17–25]. It is based on the addition of microparticles, e.g., of talc or aluminum oxide, to the growth medium. The supplemented microparticles should not be metabolized by the cells, but affect the morphological events taking place under submerged conditions, namely the agglomeration of spores, germination, and pellet development. It was previously suggested that microparticles disturb the agglomeration of spores at the initial phase of growth and thus influence pellet size [21]. The observation was made with respect to* Aspergillus niger* SKAn1015. It was recently demonstrated by Kowalska et al. [26] that different genera of filamentous fungi exhibit remarkable diversity with respect to morphological events taking place in the microparticle-containing cultures.

The biosynthesis of lovastatin, a cholesterol-lowering polyketide drug, was previously shown to be stimulated when talc microparticles were supplemented at the preculture stage [24]. This secondary metabolite belongs to the biosynthetic repertoire of* Aspergillus terreus*, a filamentous fungus employed in the biomanufacturing of lovastatin. The pellets of* A. terreus* were previously observed to be smaller in the cultures with microparticles relative to the controls and, importantly, the recorded pellet size reduction was accompanied by the elevated product titers [24]. Surprisingly, despite the industrial importance of lovastatin, the impact of other microparticles (e.g., aluminum oxide) on lovastatin biosynthesis by* A. terreus* has not been examined so far. The present study, however, was not designed to address the effectiveness of various types of microparticles in the microparticle-enhanced cultivation of* A. terreus*, but rather to suggest and evaluate an alternative approach of engineering* A. terreus* morphology, namely, the application of aluminum oxide (Al_2_O_3_) nanoparticles. The main task was to determine if replacing the microparticles with their “nano” counterparts would lead to the evident morphological changes relative to the runs with microparticles. We are not aware of any previous reports encompassing such “*micro* versus* nano*” comparative evaluations for fungal cultures.

The aim of this work was to describe the morphological and bioprocess-related consequences of adding aluminum oxide nanoparticles to the precultures of* A. terreus* ATCC 20542 and critically evaluate these effects in the context of lovastatin production. The outcomes of nanoparticle supplementation were confronted with the results of standard (without Al_2_O_3_) and microparticle-enhanced submerged cultivation. To the best of our knowledge, this is a first report on the lovastatin-oriented cultivation of* A. terreus* performed in the presence of nanoparticles. The main question was whether the nanoparticle-based approach of influencing* A. terreus* morphology could be regarded as an alternative to the microparticle-enhanced cultivation and if using the term “nanoparticle-enhanced cultivation” would be justified in this context.

## 2. Materials and Methods

### 2.1. Strain and Media

*Aspergillus terreus* ATCC 20542 was used throughout the study.

The sporulation medium was as follows: agar, 30 g l^−1^; casein peptone, 5 g l^−1^; malt extract, 20 g l^−1^ [27].

The preculture medium contained the following: yeast extract, 8 g l^−1^; lactose, 10 g l^−1^; KH_2_PO_4_, 1.51 g l^−1^; NaCl, 0.4 g l^−1^; MgSO_4_·7H_2_O, 0.51 g l^−1^; biotin, 0.04 mg l^−1^; ZnSO_4_·7H_2_O, 1 mg l^−1^; Fe(NO_3_)_3_·9H_2_O, 2 mg l^−1^; 1 ml l^−1^ of trace elements solution containing MnSO_4_, 50 mg l^−1^; Na_2_B_4_O_7_ ·10H_2_O, 100 mg l^−1^; Na_2_MoO_4_· 2H_2_O, 50 mg l^−1^; CuSO_4_·5H_2_O, 250 mg l^−1^ [24].

The production medium was the same as the preculture medium but contained less yeast extract (4 g l^−1^) and more lactose (20 g l^−1^) [24].

The pH value of all media was adjusted to 6.5 with the use of NaOH solution prior to sterilization. The media were sterilized for 20 min at 121°C.

Aluminum oxide (Al_2_O_3_) nano- or microparticles (Sigma Aldrich, USA) were sterilized separately and added to the preculture medium (6 or 12 g l^−1^) together with fungal spores at the time of inoculation. In the case of experimental controls no Al_2_O_3_ was added. According to the manufacturer's specification, the size of Al_2_O_3_ nanoparticles was <50 nm, whereas the surface area was >40 m^2^/g.

### 2.2. Cultivation Conditions

*A. terreus* ATCC 20542 was inoculated onto agar slants and after 10 days of cultivation the spores were transferred into sterile liquid preculture medium together with Al_2_O_3_ micro- or nanoparticles. The precultures were propagated in shake flasks (150 ml working volume, 500 ml total volume) for 24 hours and then 7 ml of the preculture was transferred into 150 ml of sterile liquid production medium. The subsequent shake flask cultivation was conducted for 120 h at 28°C. The cultures were propagated in a rotary shaker Certomat® BS‐1 (Sartorius Stedim, Germany, formerly B. Braun Biotech International). All experiments were performed in triplicate.

### 2.3. Analytical Methods

The samples of cultivation broth were subjected to filtration. The concentration of biomass was determined on a dry weight basis. To consider the presence of aluminum oxide particles, the biomass concentration values were corrected by subtracting the mass of aluminum oxide particles that was expected to be transferred from the preculture to production medium. The concentration values of lovastatin and lactose in the filtrate were determined by using UPLC® Acquity chromatographic system (Waters, USA). Coupling of chromatographic separation with high resolution mass spectrometry (SYNAPT G2, Waters, USA) was employed to obtain the total ion chromatograms (TICs) and perform the qualitative analysis. All chemical assays were conducted as previously described [24, 28].

The microscope OLYMPUS BX53 (Olympus Corporation, Japan) equipped with OLYMPUS cellSens Dimension Desktop 1.16 software (Olympus Corporation, Japan) was used to collect the images of fungal pellets. All image-related analytical procedures were performed as previously described [26]. The definitions of projected area, elongation, and convexity parameters can be found in the previously published report [26]. Additionally, the present study involved a parameter referred to as the shape factor, predefined in the OLYMPUS cellSens Dimension Desktop 1.16 (Olympus Corporation, Japan) software as “the area relative to the area of a circle with an equal perimeter”.

## 3. Results and Discussion

Two-stage shake flask cultivations of* A. terreus* ATCC 20542 involving the 24-h precultures were performed. The nano- or microparticles of aluminum oxide were introduced to preculture flasks at the concentration of 6 or 12 g l^−1^ together with fungal spores at the time of inoculation. The effects of Al_2_O_3_ supplementation were evaluated with respect to the observed morphology and lovastatin production at 24-hour intervals.

**Figure 1:**
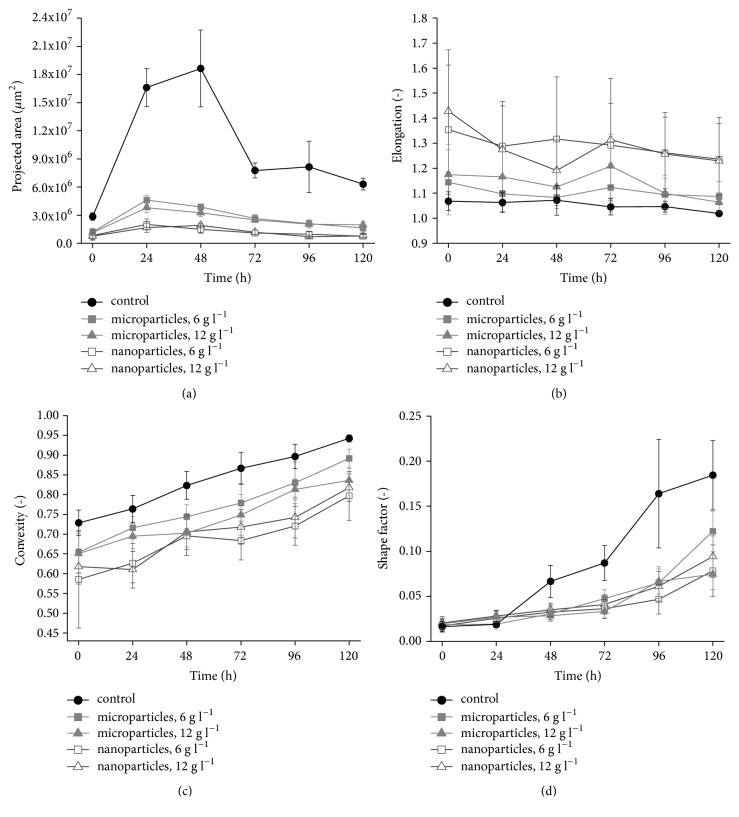
Time courses of morphological parameters calculated for* Aspergillus terreus* ATCC 20542 pellets in shake flask cultures. Projected area (a), elongation (b), convexity (c), and shape factor (d) parameters were determined for the cultures propagated with aluminum oxide (Al_2_O_3_) micro- and nanoparticles at the concentration of 6 and 12 g l^−1^ and for the control runs (without aluminum oxide). All experiments were performed in triplicate. Error bars represent the standard deviation values.

**Figure 2:**
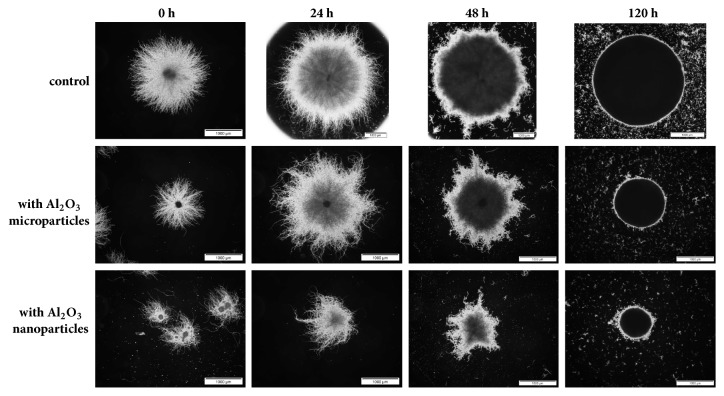
Microscopic images of* Aspergillus terreus* ATCC 20542 pellets observed in the course of shake flask cultivation in the presence of aluminum oxide (Al_2_O_3_) micro- and nanoparticles at the concentration of 6 g l^−1^. In the control no aluminum oxide was present. Presented microscopic images were taken at the time of inoculation (at 0 h) and after 24, 48, and 120 hours of growth. Scale bar: 1000 *μ*m.

The selected morphological parameters (projected area, elongation, convexity, and shape factor) of fungal mycelia were monitored throughout the cultivation by applying digital image analysis (Figure 1). The examples of microscopic images representing the tested variants are provided in Figure 2.

Generally, the pellets formed in the Al_2_O_3_-supplemented cultures were smaller than those in the control runs (Figure 1(a)). Addition of aluminum oxide led to visibly suppressed growth of pellets during the first 24 hours of cultivation. Decreased variability among projected area values was also noted as a result of Al_2_O_3_ presence, as illustrated by error bars in Figure 1(a). Notably, the values of projected area recorded for the nanoparticle-influenced variants were lower than those observed in the presence of microparticles. To be more precise, the largest values of mean projected area for the nanoparticle- and microparticle-containing cultures were equal to 2.0·10^6^ and 4.6·10^6^*μ*m^2^, respectively, while under standard conditions the mean projected area reached the value of 1.9·10^7^*μ*m^2^ (Figure 1(a)).

The decrease of pellet diameter was previously described for the microparticle-enhanced cultivation of* A. terreus* [24]. The results of the present study indicate that even greater decrease of pellet size can be achieved by applying nanoparticles. With regard to the elongation (Figure 1(b)) and convexity (Figure 1(c)) parameters, the mean values recorded for the nanoparticles variants were, respectively, higher and lower than in the microparticle-containing and control runs. What should be emphasized, however, is a relatively high variability of pellet elongation values observed in the cultures with Al_2_O_3_ particles, as indicated by the error bars in Figure 1(b). The pellets in the control runs were noticeably less diversified in this respect (Figure 1(b)). An opposite behavior was noted for the shape factor levels, where the standard cultivation seemed to be associated with greater differences observed among the tested objects (Figure 1(d)).

**Figure 3:**
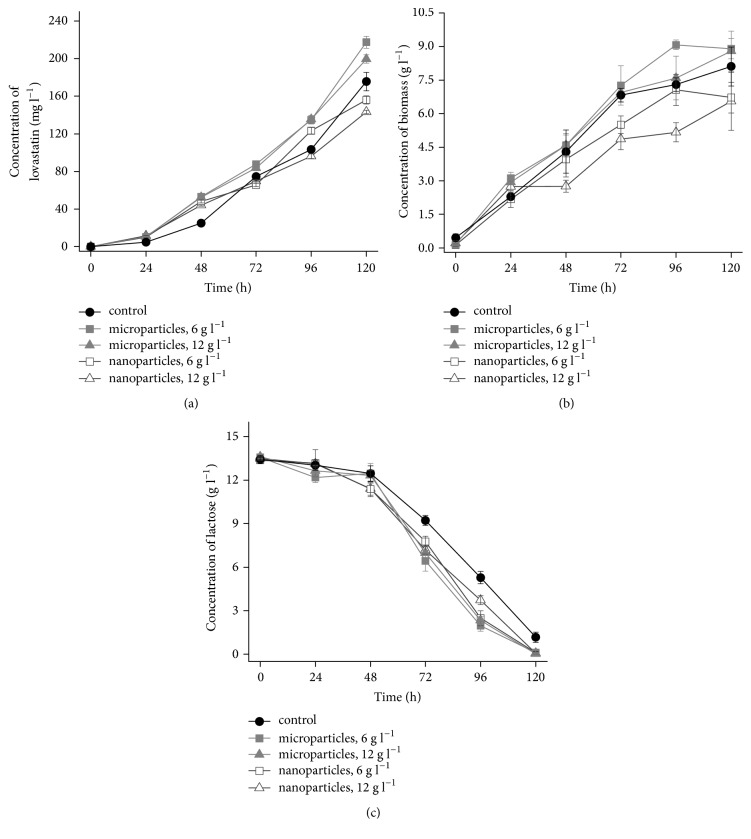
Time courses of lovastatin (a), biomass (b), and lactose (c) concentration in shake flask cultures of* Aspergillus terreus* ATCC 20542 propagated with aluminum oxide (Al_2_O_3_) micro- and nanoparticles at the concentration of 6 and 12 g l^−1^. Cultivations performed without aluminum oxide served as experimental controls. All experiments were conducted in triplicate. Error bars represent the standard deviation values.

Since the correlation between pellet size and lovastatin titers was previously described in literature [24], the morphological effects of Al_2_O_3_ supplementation recorded in the present study were regarded as highly promising. It was anticipated that nanoparticle-induced reduction of pellet size would lead to enhanced lovastatin biosynthesis. However, the results of quantitative LC-MS analysis turned out to be rather disappointing in this respect. Briefly, the decrease of the pellet size did not result in the evident increase of product titers. After 120 hours of cultivation, the final product titers in nanoparticle-containing cultures were lower than in the control and the runs with microparticles (Figure 3(a)). Earlier during the cultivation, at 96 h, the level of lovastatin at the nanoparticles concentration of 6 g l^−1^ was higher than in the control run, but this was not maintained until the end of the experiment. In contrast, the microparticle-enhanced cultivation was superior to standard runs in terms of lovastatin production not only at the end, but throughout the cultivation period. Interestingly, up to the 48 h of the process the biosynthesis of lovastatin was noticeably improved by Al_2_O_3_ particles, regardless of their size (Figure 3(a)). Then, at the 72 h of cultivation, the product levels in the nanoparticle-influenced runs were already slightly lower than in the controls, unlike in the microparticle-containing cultures. The stimulating effect of microparticles on lovastatin production was observed to be consistent until the very end of the run. This was definitely not the case in the nanoparticle-influenced cultures. Briefly, nanoparticles turned out to be effective in terms of pellet size reduction and affecting morphological forms, but not in the context of lovastatin production.

The influence of Al_2_O_3_ concentration (6 or 12 g l^−1^) on lovastatin biosynthesis was less evident in the case of the runs with microparticles than for the nanoparticle-containing cultures. In fact, until the 96 h of the run, the overlapping of product concentration profiles could be observed when the microparticles were used (Figure 3(a)). Specifically, the differences between mean lovastatin concentration values recorded at 6 or 12 g l^−1^ of Al_2_O_3_ microparticles were found to be less than 4 mg l^−1^ within the time interval between 0 and 96 h. However, at the end of the experiment (at 120 h), the mean lovastatin titer was visibly higher at 6 g l^−1^ than at 12 g l^−1^ of Al_2_O_3_ and reached 217.4 and 199.6 mg l^−1^, respectively. Notably, the time courses of lovastatin levels recorded in the presence of nanoparticles were to a greater extent affected by particles concentration. The mean titer values at 96 and 120 h were markedly lower at 12 g l^−1^ of Al_2_O_3_ than at 6 g l^−1^ (Figure 3(a)).

In the previously published work, Gonciarz and Bizukojc observed that the microparticle concentration of 12 g l^−1^ was optimal in terms of stimulating lovastatin production [24], whereas in the present study the concentration of 6 g l^−1^ turned out to be a preferred option (Figure 3(a)). It should be noted, however, that different particles were applied in these two cases. Previous experiments were conducted with the use of talc [24], whereas here aluminum oxide was employed. This is an indication that the optimal concentration of microparticles applied in the lovastatin-focused cultivation of* A. terreus* depends on the formula of the substance and need to be determined individually for each type of microparticles.

In addition to morphological parameters and lovastatin levels, the time courses of biomass (Figure 3(b)) and lactose (Figure 3(c)) concentration were monitored during the cultivation. For most of the cultivation period, the biomass concentration value was higher in the runs with microparticles than in the control. The opposite behavior was observed for the nanoparticle-containing cultures (Figure 3(b)). One may notice a correlation between final lovastatin and biomass levels recorded at 120 h. Among the tested variants, the highest biomass and lovastatin levels at 120 h were achieved in the runs with microparticles, while the lowest values corresponded to the “nano” variants. However, this correlation was not consistently observed for all time points. For example, at 48 h of cultivation, the mean biomass concentration recorded in the presence of nanoparticles was lower than in the control runs, while the reverse observation was made with respect to lovastatin levels (Figure 3(b)). Regarding lactose utilization, the Al_2_O_3_-containing cultures exhibited more rapid sugar consumption than the controls (Figure 3(c)). The final mean concentration of lactose at 120 h in the nano- and microparticle-influenced cultures did not exceed 0.1 g l^−1^, whereas in the standard runs lactose was still present at the mean concentration of 1.17 g l^−1^. Unlike in the runs with microparticles, the increased rate of lactose utilization in nanoparticle-containing cultures (Figure 3(c)) was not accompanied by the overall improvement of lovastatin production (Figure 3(a)). Here one may speculate that it was for aggravated biomass formation (less biomass ultimately grown) that lovastatin titer in the nanoparticle-influenced variants was lower than in the microparticle-enhanced cultivation.

Applying Al_2_O_3_ microparticles turned out to be beneficial in terms of product formation, whereas the presence of nanoparticles led to the decrease of final lovastatin titers. There are many factors that may have affected the biosynthetic capacity of the strain and its productivity. Importantly, the formation of lovastatin is strongly affected by oxygen availability to the cells [29]. This is associated with the underlying biosynthetic pathway, which involves a number of oxygen-dependent metabolic reactions. It is possible that the oxygen transport within nanoparticle-containing pellets is not as efficient as when microparticles are involved. This might be associated with more compact pellet structure that is likely to develop when smaller particles are applied. Nanoparticles may be portrayed as a “morphological glue” that enhances the agglomeration of spores and favors their tight packing within the pellets, but at the same hinders the aeration of mycelium by making its structure denser. Performing further analysis involving microprobes would be required to quantitatively characterize oxygen transfer within the pellets and support the hypothesis of underaeration associated with the build-up of nanoparticles.

**Figure 4:**
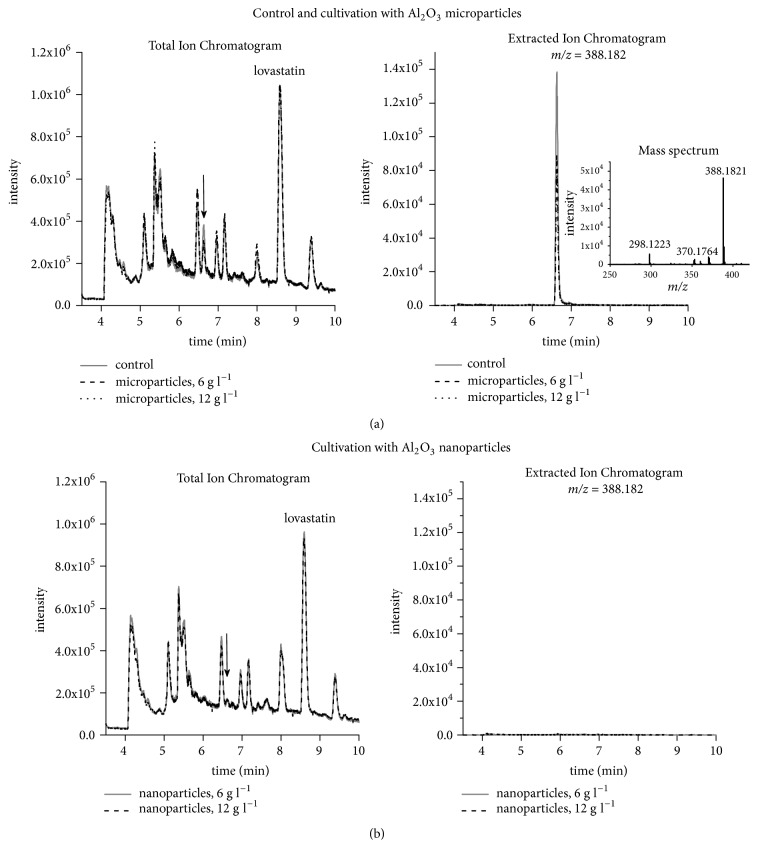
Overlay of total ion and extracted ion chromatograms (*m/z* = 388.182) obtained for experimental controls, cultures propagated with aluminum oxide (Al_2_O_3_) microparticles (a) and nanoparticles (b). Elution time corresponding to the molecule of* m/z* = 388.182 (detected in controls and cultures with Al_2_O_3_ microparticles but not in cultures with Al_2_O_3_ nanoparticles) is indicated by an arrow. Mass spectrum corresponding to this molecule is also provided (a). All analyses were performed in the ESI+ mode.

In addition to the morphological outcomes, a notable chemical effect associated with the nanoparticle-influenced cultivation was observed. The ESI+ total ion chromatograms corresponding to respective experimental variants were compared in order to determine whether the addition of aluminum oxide micro- or nanoparticles affected the chemical profile of the broth (Figure 4).

It turned out that the peaks recorded for the control runs had their counterparts in the spectrum representing the microparticle-enhanced cultivation. This was evident when the overlay of chromatograms was consulted (Figure 4(a)). Interestingly, the presence of nanoparticles had a noticeable impact on the peak at 6.6 min (indicated by an arrow in Figure 4). Due to the fact that this peak was not completely absent from the nanoparticle-related chromatogram, the manual scanning procedures were applied to determine the* m/z* values responsible for this signal. The underlying mass spectrum corresponding to the control and the runs with microparticles revealed a relatively intensive* m/z* peak at 388.1821 (Figure 4(a)). When the mass scanning of total ion chromatograms representing the nanoparticle-influenced cultures was performed, no peaks of similar* m/z* values were detected (using the* m/z* window of 0.01). Therefore, the compound eluting at t=6.6 min and corresponding to the mass spectrum presented in Figure 4(a) was detected solely in the controls and microparticle-containing variants. It should be noted that the above-described chemical outcome was dependent on the size of Al_2_O_3_ particles (“micro” or “nano”) but not on their concentration (Figure 4). It cannot be excluded that the addition of Al_2_O_3_ nanoparticles was responsible for inhibiting the biosynthetic route leading to a yet unidentified secondary metabolite of* A. terreus*. To verify this idea, the natural products database* Antibase 2014: The Natural Compound Identifier* [30] was extensively screened in an attempt to identify the underlying molecule, under an assumption that the peak at* m/z*=388.1821 represented the pseudomolecular [M+H]^+^ ion. However, this attempt proved to be unsuccessful and the structure of the substance remains unknown. Further chemical studies are necessary to discover its origin and identity.

**Figure 5:**
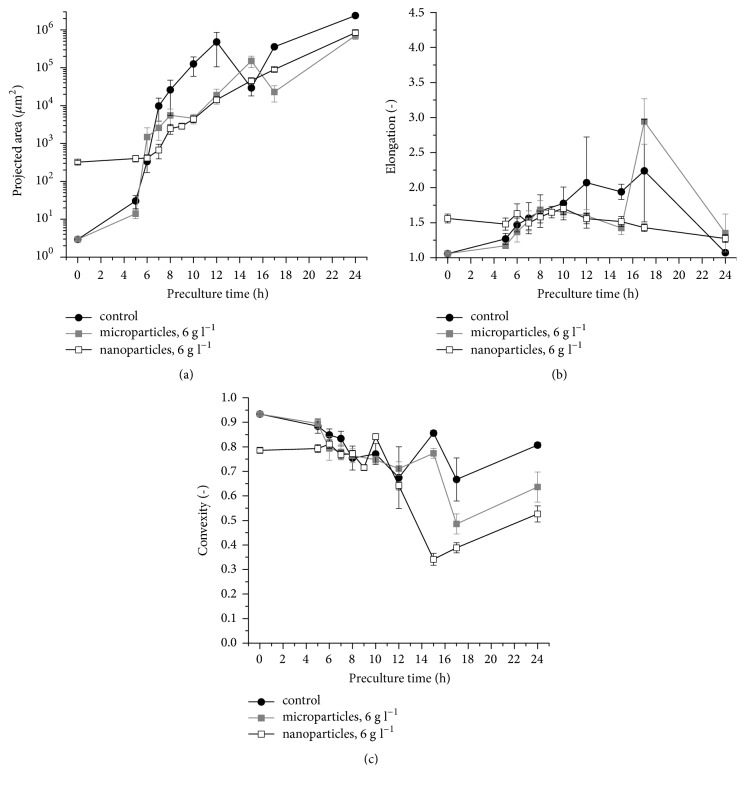
Time courses of morphological parameters calculated for* Aspergillus terreus* ATCC 20542 precultures. Projected area (a), elongation (b), convexity (c), parameters were determined for the shake flask precultures propagated with aluminum oxide (Al_2_O_3_) nanoparticles at the concentration of 6 g l^−1^. The data for experimental control (preculture without aluminum oxide) and the runs with Al_2_O_3_ microparticles at the concentration of 6 g l^−1^ are taken from the study of Kowalska et al. [26]. All experiments were performed in triplicate. Error bars represent the standard deviation values.

To gain further insights into the nanoparticle-influenced cultivation of* A. terreus*, the scope of the experiments was expanded to include the quantitative morphological characterization of the preculture phase. A report describing the events occurring in the preculture of both standard and microparticle-enhanced cultivation of selected fungal species (including* A. terreus*) was published recently by Kowalska et al., but the influence of nanoparticles was not examined by the authors [26]. The data gathered in the present study complements the previous findings regarding the morphological scenarios in submerged fungal cultures. Specifically, the characterization of the preculture phase was based on three morphological parameters, namely, the projected area (Figure 5(a)), elongation (Figure 5(b)), and convexity (Figure 5(c)). As these parameters were previously determined in the above-mentioned work of Kowalska et al. [26] for the standard and microparticle-containing precultures of* A. terreus*, the comparison of different variants was possible (Figure 5).

**Figure 6:**
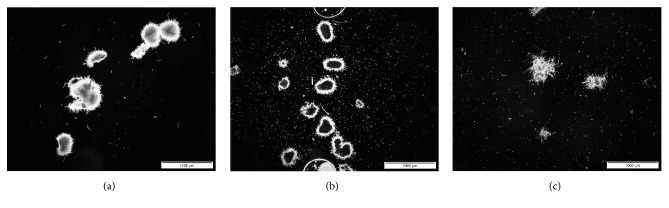
Microscopic images of* Aspergillus terreus* ATCC 20542 taken in the course of shake flask preculture without Al_2_O_3_ (a), in the presence of Al_2_O_3_ micro- (b) and nanoparticles (c) at the concentration of 6 g l^−1^. Presented microscopic images were taken after 15 h of cultivation. Scale bar: 1000 *μ*m.

The examples of microscopic images of fungal structures observed in the examined runs are provided in Figure 6.

Notably, in contrast to the Al_2_O_3_ microparticles, the nanoparticles exhibited agglomeration from the very start of the preculture. This behavior was reflected by the noticeable differences of projected area, elongation, and convexity values just after introducing the spores into the media (0 h of preculture) compared to the ones obtained for the microparticle-enhanced and standard cultivations (Figure 5). The mean value of projected area of the observed nanoparticle agglomerates at the onset of the preculture was equal to 320 *μ*m^2^ (Figure 5(a)). In the case of the microparticle-based variant, the agglomerates of similar projected area were developed between the 5 and 6 h of the run (Figure 5(a)). Interestingly, at the 15 h of cultivation, the mean value of convexity parameter was at the level of 0.34, which was the lowest convexity value obtained throughout the preculture-related experiments, noticeably lower than for the run with microparticles and the standard cultivation. As the cultivation proceeded, between the 15 and 24 h of the preculture, the level of convexity observed in the presence of nanoparticles remained lower than the values observed for the microparticle-enhanced and standard runs (Figure 5(c)). As far as the elongation parameter is concerned, the mean values determined over the course of the nanoparticle-influenced preculture remained within the relatively narrow interval between 1.2 and 1.7, whereas in the standard and microparticle-containing precultures the recorded intervals were from 1.0 to 2.3 and from 1.0 to 2.9, respectively (Figure 5(b)). Furthermore, the development of multi-core agglomerates was typically observed in the presence of nanoparticles in the preculture, giving rise to multi-core pellets (Figure 6(c)).

Among the available nanoparticles, the Al_2_O_3_ nanopowder was chosen to be applied in this work. This decision was greatly influenced by the study of Etschmann et al. [31], who examined the release of ions to the liquid as a result of microparticle addition and leaching. According to the reported results, Al_2_O_3_ could be considered as an inert substance compared to other tested products due to the relatively low level of particle leaching. However, the study was focused on microparticles, not on their “nano” counterparts. Moreover, it should be noted here the Al_2_O_3_ nanoparticles were previously investigated with regard to their potential toxicity. For example, Sadiq et al. [32] described the toxicity exhibited by Al_2_O_3_ nanoparticles towards microalgae species* Scenedesmus* sp. and* Chlorella* sp. isolated from aquatic environment. The interactions of the nanoparticles with the cell surface were reported to be possibly associated with the toxicity in this case [32]. Al_2_O_3_ nanoparticles were also shown to induce oxidative stress in rats [33], cause DNA damage in mammalian cell lines [34], and trigger the processes of programmed cell death in plant cells [35]. In the light of the previous findings, it cannot be excluded that Al_2_O_3_ displays toxicity towards* A. terreus* and thus inhibits the growth of pellets and the production of lovastatin. However, further research is required to verify these speculations. Future efforts should determine the correlation between the toxicity levels (if any), the concentration of Al_2_O_3_, and the impact on fungal morphology.

All in all, the approach of using the Al_2_O_3_ nanoparticles as an alternative to its “micro” counterpart turned out to be effective in morphological terms, since it truly did lead to altered growth characteristics relative to controls and the runs with microparticles, as illustrated by the parameter values depicted in Figure 1. However, the anticipated improvement with regard to lovastatin production was not observed as a consequence of these changes. Therefore, in a bioprocess perspective, using the term “nanoparticle-enhanced cultivation” is definitely not justified in this case. It does not mean, however, that in the light of these results the application of nanoparticles in fungal cultivations should be perceived as irrelevant. We believe that further studies are required to gather more process-related data and examine this method in a broad perspective. Preferably, future efforts should be focused on bioreactor experiments, as it is well-known that mixing and aeration conditions are of great importance whenever a desired morphological form is to be achieved [12–15]. Furthermore, the present study was based on the standard growth medium typically applied in lovastatin production [24]. The reason for that was to present the outcomes of nanoparticle-involving experiment in the comparative perspective with the results of microparticle-enhanced cultivation and not to study the influence of medium composition on lovastatin production. Possibly, the results of the future studies, conducted with the use of different media, cultivation strategies, and resources, will provide novel insights into the nanoparticle-based cultivation.

## 4. Conclusions

The following conclusions can be formulated on the basis of the results of the present study:

1. The pellets of* A. terreus* ATCC 20542 developed in the presence of aluminum oxide nanoparticles are smaller, more elongated, and less convex than in the corresponding microparticle-enhanced cultivations.
2. In the context of lovastatin biosynthesis by* A. terreus* ATCC 20542, the morphological engineering approach involving Al_2_O_3_ nanoparticles supplementation is not sufficiently effective to be perceived as an alternative to the microparticle-enhanced cultivation, despite its evident morphological impact.
3. The concentration of biomass in the nanoparticle-containing cultures does not reach the level noted in the microparticle-enhanced cultivation, what may be one of the reasons responsible for relatively low product titers.
4. There are differences with regard to chemical profiles exhibited by nano- and microparticle-influenced submerged cultures of* A. terreus* ATCC 20542.

## Data Availability

The data used to support the findings of this study are included within the article.
